# Machine learning for the prediction of cognitive impairment in older adults

**DOI:** 10.3389/fnins.2023.1158141

**Published:** 2023-04-27

**Authors:** Wanyue Li, Li Zeng, Shiqi Yuan, Yaru Shang, Weisheng Zhuang, Zhuoming Chen, Jun Lyu

**Affiliations:** ^1^Department of Rehabilitation, The First Affiliated Hospital of Jinan University, Guangzhou, Guangdong, China; ^2^The Second Clinical Medical College of Guizhou University of Traditional Chinese Medicine, Guiyang, Guizhou, China; ^3^Department of Neurology, The First Affiliated Hospital of Jinan University, Guangzhou, Guangdong, China; ^4^Department of Rehabilitation, Henan Provincial People's Hospital, People's Hospital of Zhengzhou University, Zhengzhou, Henan, China; ^5^Department of Clinical Research, The First Affiliated Hospital of Jinan University, Guangzhou, Guangdong, China; ^6^Guangdong Provincial Key Laboratory of Traditional Chinese Medicine Informatization, Guangzhou, Guangdong, China

**Keywords:** cognitive function, NHANES, older adults, machine learning, prediction model

## Abstract

**Objective:**

The purpose of this study was to develop and validate a predictive model of cognitive impairment in older adults based on a novel machine learning (ML) algorithm.

**Methods:**

The complete data of 2,226 participants aged 60–80 years were extracted from the 2011–2014 National Health and Nutrition Examination Survey database. Cognitive abilities were assessed using a composite cognitive functioning score (Z-score) calculated using a correlation test among the Consortium to Establish a Registry for Alzheimer's Disease Word Learning and Delayed Recall tests, Animal Fluency Test, and the Digit Symbol Substitution Test. Thirteen demographic characteristics and risk factors associated with cognitive impairment were considered: age, sex, race, body mass index (BMI), drink, smoke, direct HDL-cholesterol level, stroke history, dietary inflammatory index (DII), glycated hemoglobin (HbA1c), Patient Health Questionnaire-9 (PHQ-9) score, sleep duration, and albumin level. Feature selection is performed using the Boruta algorithm. Model building is performed using ten-fold cross-validation, machine learning (ML) algorithms such as generalized linear model (GLM), random forest (RF), support vector machine (SVM), artificial neural network (ANN), and stochastic gradient boosting (SGB). The performance of these models was evaluated in terms of discriminatory power and clinical application.

**Results:**

The study ultimately included 2,226 older adults for analysis, of whom 384 (17.25%) had cognitive impairment. After random assignment, 1,559 and 667 older adults were included in the training and test sets, respectively. A total of 10 variables such as age, race, BMI, direct HDL-cholesterol level, stroke history, DII, HbA1c, PHQ-9 score, sleep duration, and albumin level were selected to construct the model. GLM, RF, SVM, ANN, and SGB were established to obtain the area under the working characteristic curve of the test set subjects 0.779, 0.754, 0.726, 0.776, and 0.754. Among all models, the GLM model had the best predictive performance in terms of discriminatory power and clinical application.

**Conclusions:**

ML models can be a reliable tool to predict the occurrence of cognitive impairment in older adults. This study used machine learning methods to develop and validate a well performing risk prediction model for the development of cognitive impairment in the elderly.

## Introduction

Cognitive impairment is a process of neurodegenerative aging that begins with mild cognitive impairment and ends with severe dementia (McKhann et al., 2011; Peng et al., 2020). Cognitive impairment manifests as impairment in multiple functions: communication and language, attention, memory, reasoning, judgment, and visual perception (McKhann et al., 2011). As medical advances continue to extend human life expectancy, cognitive decline associated with aging is a growing public health problem, with approximately one in nine older adults suffering from cognitive impairment, which can have a significant impact on their work and lives, even in its mild stages (Johansson et al., 2015). There is no cure for cognitive impairment, so early prevention and early intervention are important to delay its onset (Barnes and Yaffe, 2011).

The Dietary Inflammatory Index (DII) combines the anti-/proinflammatory effects of multiple food components and is a recognized indicator of overall dietary inflammation (Ryu et al., 2019). There is evidence that both DII and age are negatively associated with cognitive function (Frith et al., 2018). However, there are many risk factors for cognitive impairment in older adults, such as race, nutritional status, and a history of chronic disease (Hugues et al., 2021; Yeh et al., 2022), and these factors cannot be ignored if cognitive impairment is to be accurately predicted using clinical models, which are tools that combine multiple key factors to predict specific outcomes (Zhang et al., 2018). Two recent studies used generalized linear mixed models to demonstrate that urban environmental features, such as the percentage of commercial land in residential areas, can positively affect cognitive functions such as working memory and processing speed by promoting engagement in physical activity (Cerin et al., 2021), and the associated negative effects of higher levels of ambient air pollution (Cerin et al., 2022).

Some multivariate prediction models based on traditional statistical methods, such as logistic regression (LR) and Cox proportional risk models (Yue et al., 2022), have been developed for the occurrence of cognitive impairment in older adults (Xie et al., 2021). Although previous studies have been useful to better understand the relationship between environment and cognition at the population level, the relationship between individual variables in the clinical setting is complex and LR, which deals with linear relationships between independent and dependent variables by default, may oversimplify the complex non-linear relationships. In addition, LR is susceptible to multicollinearity among variables, which may reduce the performance of the model. Therefore, exploring more effective and accurate prediction tools is extremely important for the management of elderly patients with cognitive impairment.

In recent years, machine learning (ML) has attracted the attention and recognition of clinicians due to the development of statistical theory and computer technology. Novel ML techniques have been widely used in predictive models for various diseases and have shown better performance compared to traditional predictive models. Recent studies have used machine learning to predict cognitive decline (Pinheiro et al., 2019) and the future incidence of Alzheimer's disease (Hu et al., 2021), using population-level sociodemographic and health data. However, there are significant gaps in our understanding of the models and factors that apply to predict specific domains of cognitive function in middle-aged and older adults. This study aims to address this gap by comparing the performance of five different machine learning models. To achieve this goal, we extracted demographics, lifestyle, nutrition, physical inflammation, and blood lipids together as variables to analyze a large population-representative sample of older adults for early prediction of the risk of new-onset cognitive impairment in older patients, attempted to develop and validate multiple ML models to predict the risk of cognitive impairment in older adults and found the model with the best predictive performance.

## Methods

### Data source

We conducted a cross-sectional study of data from the National Health and Nutrition Examination Survey (NHANES) public database of the United States. An informed-consent form has been signed for all participants in the database either by themselves or by a proxy. This database is comprehensive, accurate, and systematic, and provides a wealth of data for use in developing policies on nutrition and public health (Wu et al., 2021). There is a dedicated system management system responsible for NHANES data collection and updating, and the survey data are updated regularly on the website and are open to access by the public free of charge.

### Participants

**Figure 1:**
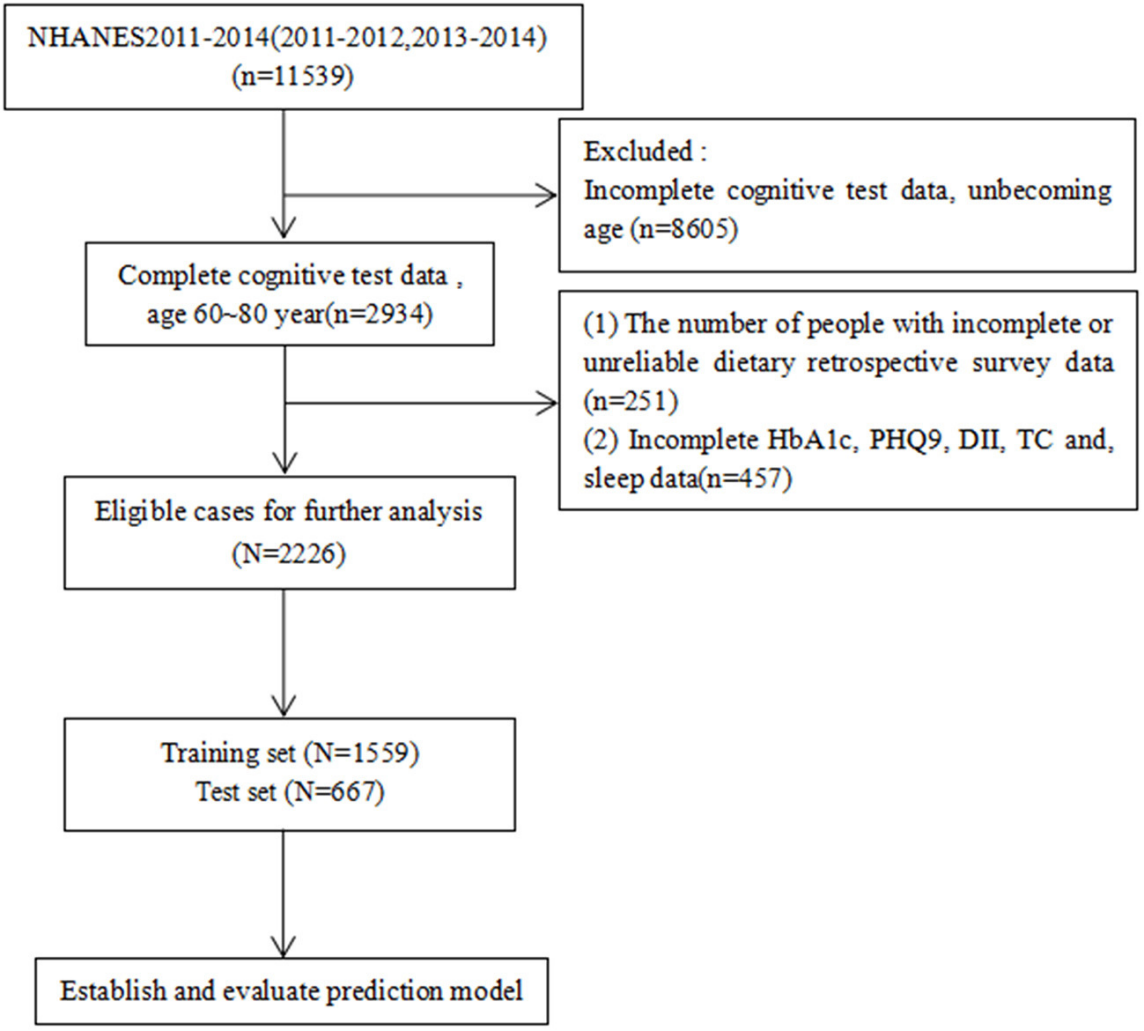
Case inclusion process. DII, Dietary Inflammatory Index; HbA1c, glycated hemoglobin; PHQ9, Patient Health Questionnaire-9; sleep, how much sleep do you get; HDL, direct HDL-cholesterol level.

Data were obtained from NHANES database for the years 2011–2014. We included four cognitive assessment tests to calculate the composite cognitive functioning score (Z-score): age, sex, race, body mass index (BMI), drink, smoke, direct HDL-cholesterol level, stroke history, DII, glycated hemoglobin (HbA1c), Patient Health Questionnaire-9 (PHQ-9) score, sleep duration, and albumin level (Yang et al., 2020). Only people aged 60–80 years were included, and data with missing values were excluded; we only analyzed complete data, and 2,226 participants were recruited for this study after screening using the exclusion criteria (Figure 1).

### Depressive symptoms

The Patient Health Questionnaire (PHQ) is a depression screening scale. The answer categories of the nine items “none at all,” “a few days,” “more than half of the days,” and “almost every day” were given a score ranging from 0 to 3. There are nine projects, with a maximum score of 27 points.

### Calculation of DII

This study analyzed 28 of the 45 food components from the original DII: carbohydrate, protein, total fat, alcohol, fiber, cholesterol, saturated fat, MUFA, PUFA, n-3 fatty acids, n-6 fatty acids, niacin, vitamin A, thiamin (vitamin B1), riboflavin (vitamin B2), vitamin B6, vitamin B12, vitamin C, vitamin D, vitamin E, Fe, Mg, zinc, selenium, folic acid, beta-carotene, caffeine, and energy. There is evidence that DII is still useful for predicting overall inflammation when only information on fewer food components is available (Shivappa et al., 2014a). The calculation of the DII is based on a 24-h dietary recall interview or food records (Shivappa et al., 2014b; Wirth et al., 2017). There are standard reference values for each food parameter in the world database. The 24-h dietary recall data were multiplied by standard food parameters from the world database to obtain individual dietary inflammation composite cognitive function scores (Z-scores) relative to the standard global average. We transformed this value into a percentile to reduce bias. Each percentile was doubled, and then 1 was subtracted from it. The percentage values for each food parameter were then multiplied by their respective “overall food parameter-specific inflammatory effect scores” to obtain individual food-specific DII scores. Finally, the DII scores for all individual food components were summed to obtain the “overall DII score” for each person (Shivappa et al., 2014a).

### Cognitive function

Cognitive function was assessed using four tests that were administered in the following order: the Consortium to Establish a Registry for Alzheimer's Disease Word Learning (CERAD-WL) test, the Animal Fluency (AF) test, the Digit Symbol Substitution Test (DSST), and the CERAD Delayed Recall (CERAD-DR) test. The CERAD-WL and CERAD-DR tests include three sequential learning test phases and a delayed-recall test phase (Rosen, 1983). The maximum score on the CERAD-WL test was 30 points, and that of the CERAD-DR test, which was performed after the AF test and DSST, was 10 points. In the AF test (Clark et al., 2009), participants were asked to name as many animals as possible in 1 min, and received 1 point for each correct answer. This test examined the absolute verbal fluency and executive function of the participants. For the DSST (Brody et al., 2019), we asked participants to copy the corresponding symbols into the 133 boxes next to the numbers within 2 min, with correct sets earning 1 point to give a maximum of 133 points. This test examined attention and memory functions.

The composite cognitive score (Z-score) was calculated. To exclude uneven differences in individual cognitive scores, we used a Z-score that consisted of the CERAD-WL test, CERAD-DR test, AF test, and DSST as the total globally standardized cognitive function score. The Z-score was calculated as *Z* = (*x-u*)/σ, where x is the raw score, *u* is the population mean, and σ is the population standard deviation. A Z-score of < -1 was considered to indicate that the person had cognitive impairment (Wirth et al., 2017; Frith et al., 2018).

### Statistical analysis

We calculated new sample weights for the data analysis (Liu et al., 2013). Continuous variables that did not conform to a normal distribution are expressed as median (interquartile range) values, with mean and standard-error values provided for the other continuous variables. Intergroup comparisons of baseline data were performed using weighted-sample independent *t*-tests for continuous variables and chi-square tests for categorical variables. Feature selection is an important step in model construction. The Boruta algorithm is used to identify the most important features by comparing the Z-value of each feature with the Z-value of the “shadow feature”. The Z-value of each attribute is obtained from the Random Forest model at each iteration by replicating all the true features and disrupting them in order, and the Z-value of the shadow is created by randomly disrupting the true features. A true feature is considered “significant” if its Z-value is greater than the maximum Z-value of the shaded feature across multiple independent trials (Lei et al., 2021).

After feature selection, five ML algorithms, generalized linear model (GLM), random forest (RF), support vector machine (SVM), artificial neural network (ANN), and stochastic gradient boosting (SGB) are used for model construction. The data set was randomly divided into a training set and a test set using the accepted Pareto principle (70-30 partition). The training set contains 70% of the observations used for model selection and tuning. Ten-fold cross-validation is used for cross-validation during the training period, the training set data set is divided into 10 copies, 9 copies are used as the training set and 1 copy is used as the validation set in turn, and finally, the average of the accuracy of the algorithm obtained 10 times is taken as the accuracy of the algorithm. Thirty percentage of the original dataset was used as a test set to evaluate the model. The test set results are used for model performance evaluation. In our cases, the model with the highest area under the curve (AUC) of the receiver operating characteristic (ROC) curve was selected as the best model for each algorithm. The performance of prediction models was performed in terms of discrimination and clinical utility. The discriminative performance of the five models was quantitatively evaluated by ROC curves of under the curve, specificity, sensitivity, accuracy, and specificity/sensitivity. Clinical applications are studied through decision-curve analysis (DCA). Results for which *p* < 0.05 were considered significant. All analyses were performed using R software (version 4.0.2).

## Results

### General characteristics

The 2,226 participants included 384 with cognitive impairment. Table 1 describes the differences in characteristics between the cognitively impaired and non-cognitively impaired groups. The age of the cognitively impaired group was significantly higher than that of the non-cognitively impaired patients. Non-Hispanic whites accounted for the majority of non-cognitively impaired patients (82.94%). A higher percentage of people in the non-cognitively impaired group drank alcohol (74.67%). The stroke rate was higher in the cognitive impairment group (12.82%). DII, HbA1c, and PHQ9 were significantly higher in the cognitively impaired group than in the non-cognitively impaired patients. Albumin was significantly lower in the cognitively impaired group than in the non-cognitively impaired patients. The general characteristics of the participants are listed in Table 1.

**Table 1:** Characteristics of participants.

	**Non-cognitive impairment (*n* = 1,842)**	**Cognitive impairment (*n* = 384)**	***P*-value**
Age^a^ (year)	68.556 (0.220)	73.466 (0.601)	< 0.0001^**^
Sex^b^, *n* (%)			0.87
Male	912 (47.10)	218 (46.49)	
Female	930 (52.90)	166 (53.51)	
Race^b^, *n* (%)			< 0.0001^**^
Mexican American	127 (2.56)	53 (9.72)	
Non-Hispanic White	1,014 (82.94)	111 (53.01)	
Non-Hispanic Black	380 (6.54)	126 (20.45)	
Other	321 (7.95)	94 (16.82)	
Body mass index^a^, (kg/m^2^)	28.991 (0.259)	28.128 (0.634)	0.261
TC	1.434 (0.020)	1.399 (0.040)	0.456
Smoke at least 100 cigarettes in life^b^, *n* (%)			0.48
Yes	922 (49.55)	208 (52.12)	
No	920 (50.45)	176 (47.88)	
Had at least 12 alcohol drinks/year^b^, *n* (%)			< 0.0001^**^
Yes	1,307 (74.67)	244 (58.89)	
No	535 (25.33)	140 (41.11)	
Ever told you had stroke^b^, *n* (%)			< 0.001^**^
Yes	91 (4.45)	49 (12.82)	
No	1,751 (95.55)	335 (87.18)	
DII^a^	1.265 (0.084)	2.027 (0.107)	< 0.0001^**^
HbA1c^a^	5.900 (0.033)	6.264 (0.103)	0.003^**^
PHQ9^a^	2.541 (0.121)	4.471 (0.394)	< 0.0001^**^
Sleep^a^, (h)	7.128 (0.030)	7.168 (0.079)	0.676
Albumin^a^, (g/L)	42.324 (0.103)	41.137 (0.235)	< 0.0001^**^

^a^Values are mean ± (standard error) [Mean ± (SE)].

^b^Chi-square test was used to compare the difference between normal and low cognitive function participants, data is number of subjects (percentage) or medians (interquartile ranges).

DII, Dietary Inflammatory Index; HbA1c, glycated hemoglobin; PHQ9, Patient Health Questionnaire-9; sleep, how much sleep do you get.

^**^P < 0.01.

### Feature selection

**Figure 2:**
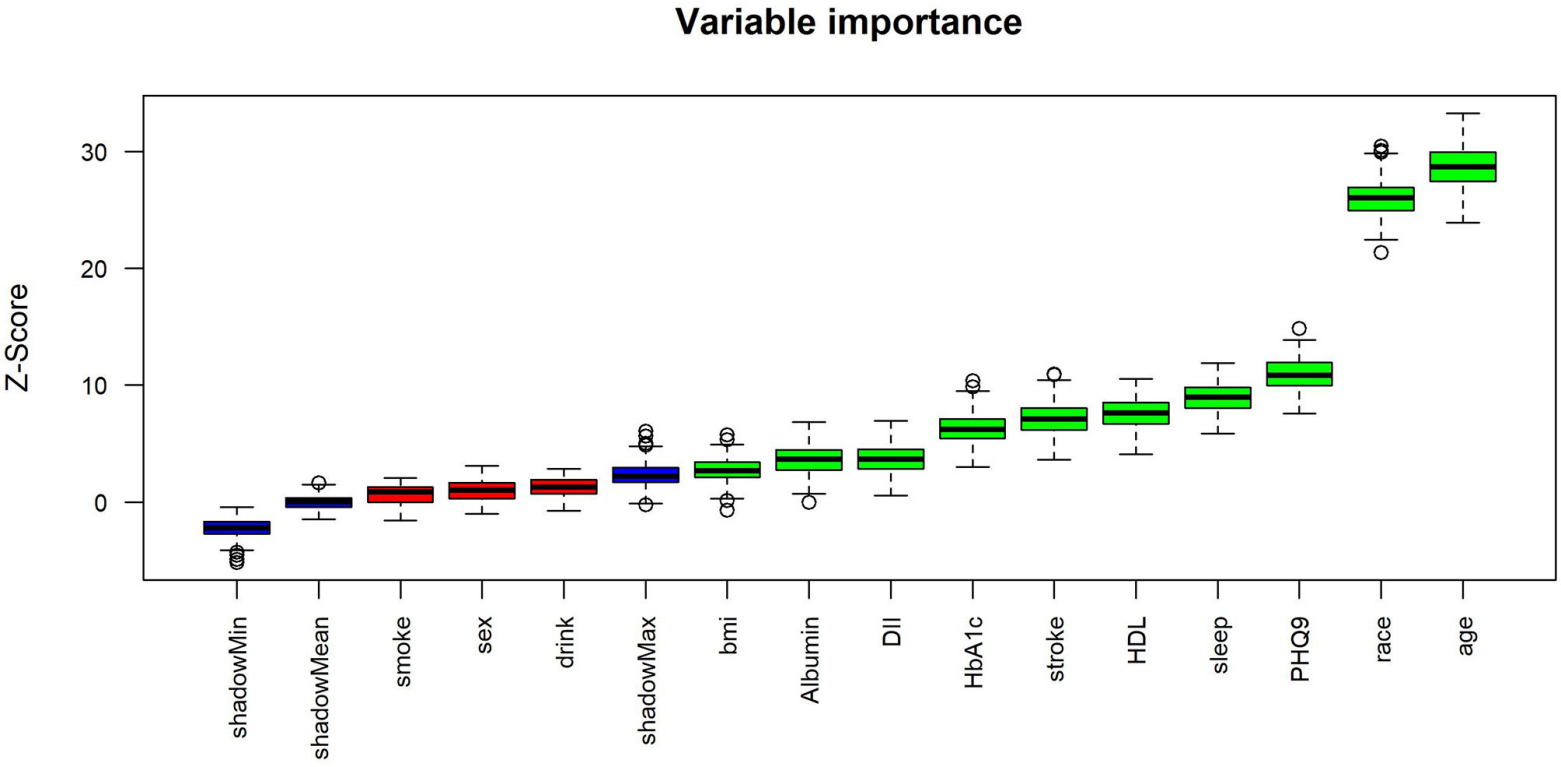
Feature selection based on the Boruta algorithm. bmi, Body mass index; drink, Had at least 12 alcohol drinks/year; smoke, Smoke at least 100 cigarettes in life; stroke, ever told you had a stroke; DII, Dietary Inflammatory Index; HbA1c, glycated hemoglobin; PHQ9, Patient Health Questionnaire-9; sleep, how much sleep do you get.

The results of feature screening based on Boruta algorithm are shown in Figure 2. The 10 variables most strongly associated with cognitive impairment, in order of z-value, were age, race, body mass index (BMI), direct HDL-cholesterol level, stroke history, dietary inflammatory index (DII), glycated hemoglobin (HbA1c), Patient Health Questionnaire-9 (PHQ-9) score, sleep duration, and albumin level.

### Model performance comparison

**Figure 3:**
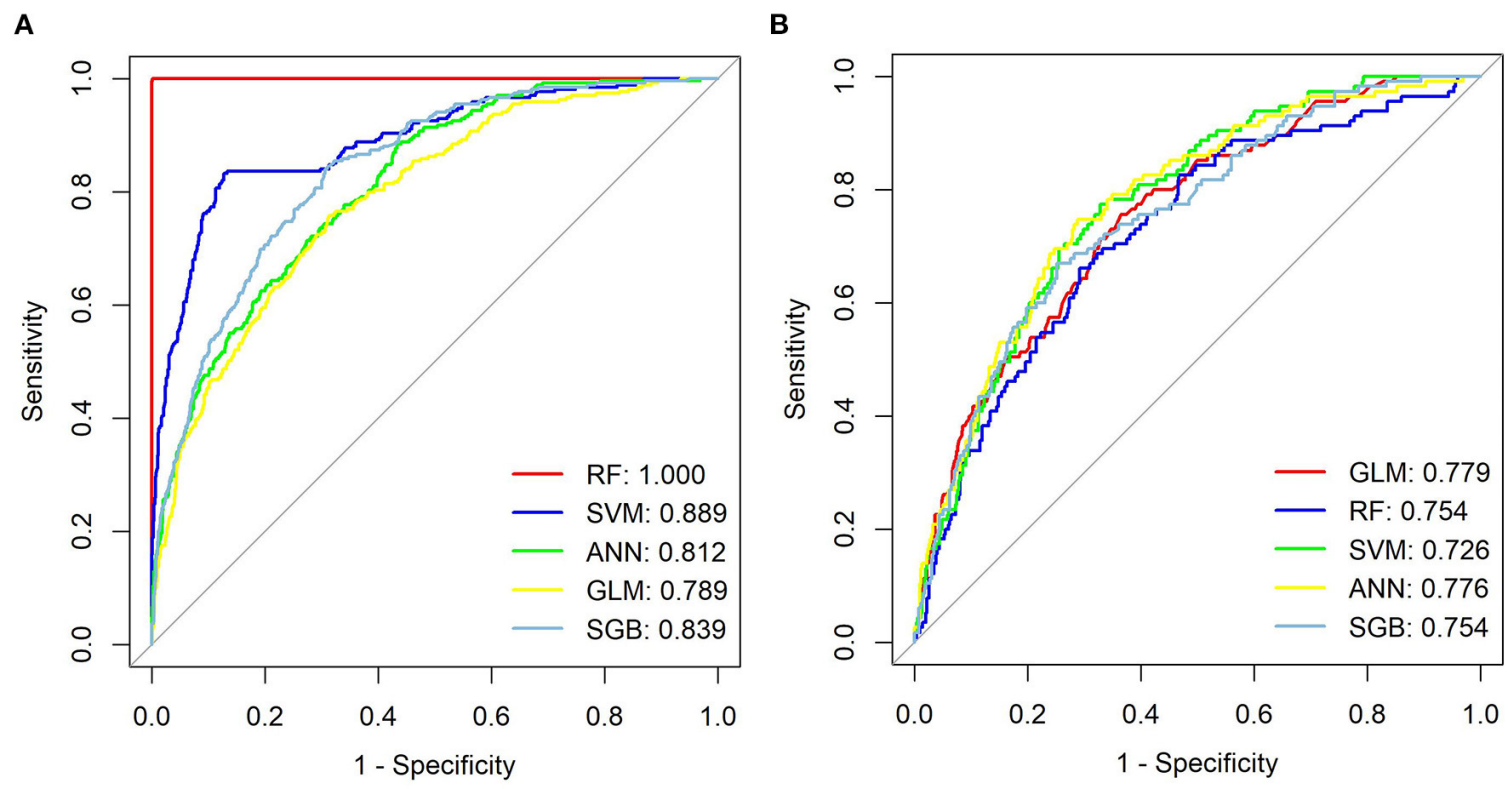
ROC curves from seven models, training set **(A)** and test set **(B)**. GLM, generalized linear model; RF, Random Forest; SVM, support vector machine; ANN, artificial neural network; SGB, Stochastic Gradient Boosting.

**Figure 4:**
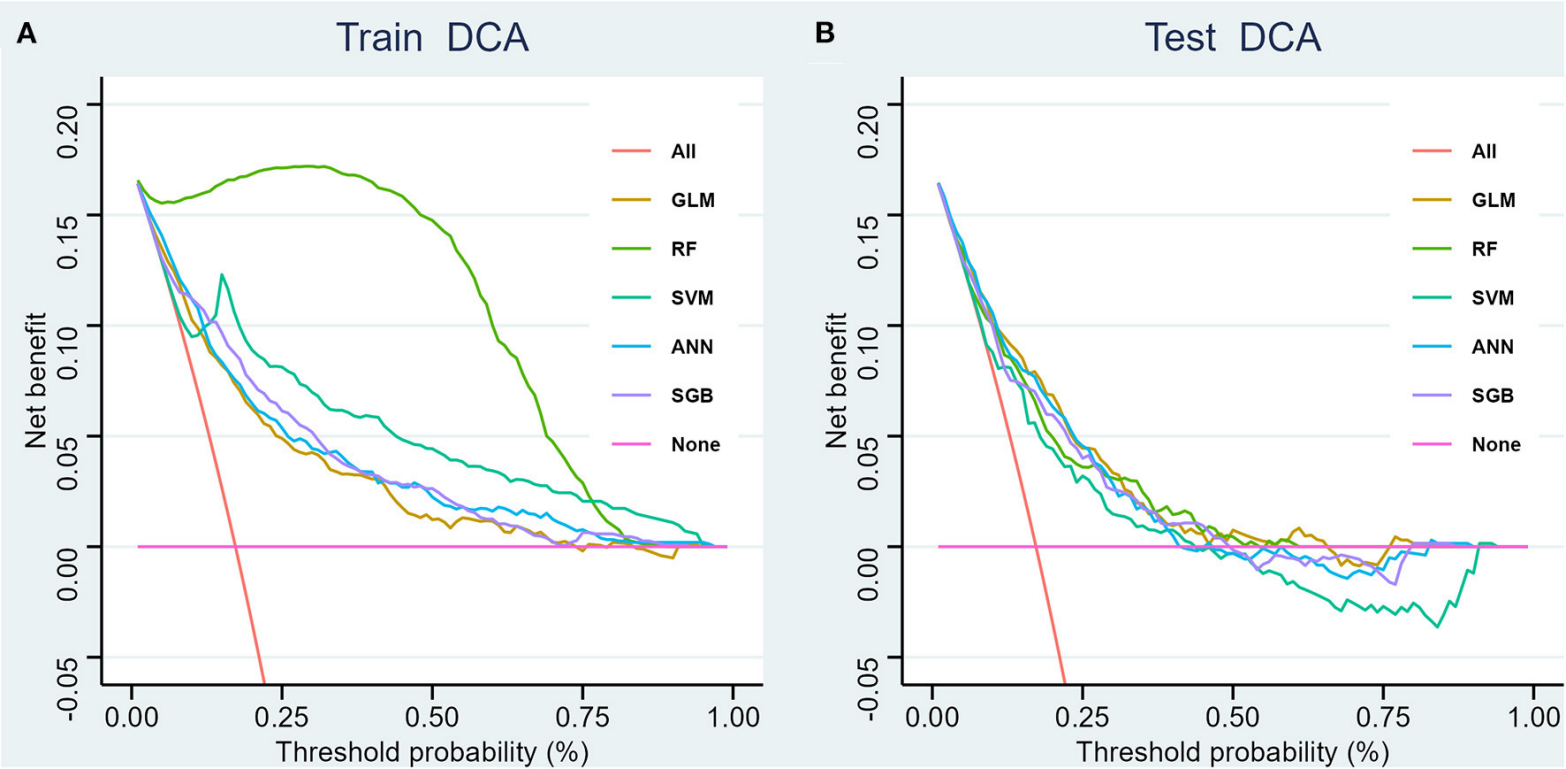
Decision curve analysis for four models, training set **(A)** and test set **(B)**. GLM, generalized linear model; RF, Random Forest; SVM, support vector machine; ANN, artificial neural network; SGB, Stochastic Gradient Boosting.

We generated five ML models to predict the probability of cognitive impairment in older adults. Figure 3 shows the discriminant performance of the five models for the training set and the test set in terms of ROC curves. The test set of our model, Figure 3B, shows that among the five models, the GLM model (AUC = 0.779) has the best prediction effect on cognitive impairment in older adults, followed by the ANN (AUC = 0.776), SGB (AUC = 0.754), RF (AUC = 0.754), and SVM (AUC = 0.726) models. When using the GLM model (AUC = 0.779) as a reference, ANN, RF, SGB, and SVM were inferior in predicting cognitive impairment in older adults. Table 2 lists a detailed set of performance indicators for the five models. In the test set, the ROC curve shows that the GLM model has better prediction performance, with an area under the curve of 0.779. The SGB model has the highest specificity and accuracy, with values of 0.748 and 0.735, respectively. The ANN model has a higher sensitivity, with a value of 0.774. Figures 4A, B show the DCA curves of the training set and the test set, respectively. According to the DCA curves of the test set (Figure 4B), the GLM model exhibits a greater net income sum compared to other models, indicating that the GLM model is the optimal model with good clinical utility.

**Table 2:** Model performance metrics.

**Models**	**Area under the curve**	**Optimal cutoff**	**Specificity**	**Sensitivity**	**Accuracy**	**Specificity/sensitivity**
GLM train	0.789	0.159	0.688	0.758	0.7	0.908
GLM test	0.779	0.174	0.712	0.748	0.718	0.952
RF train	1	0.305	0.999	1	0.999	0.999
RF test	0.754	0.153	0.636	0.757	0.657	0.840
SVM train	0.889	0.149	0.873	0.833	0.866	1.048
SVM test	0.726	0.154	0.708	0.661	0.7	1.071
ANN train	0.812	0.115	0.567	0.885	0.622	0.641
ANN test	0.776	0.179	0.672	0.774	0.69	0.868
SGB train	0.839	0.146	0.694	0.84	0.719	0.826
SGB test	0.754	0.178	0.748	0.67	0.735	1.116

GLM, generalized linear model; RF, Random Forest; SVM, support vector machine; ANN, artificial neural network; SGB, Stochastic Gradient Boosting.

### Variable importance

**Figure 5:**
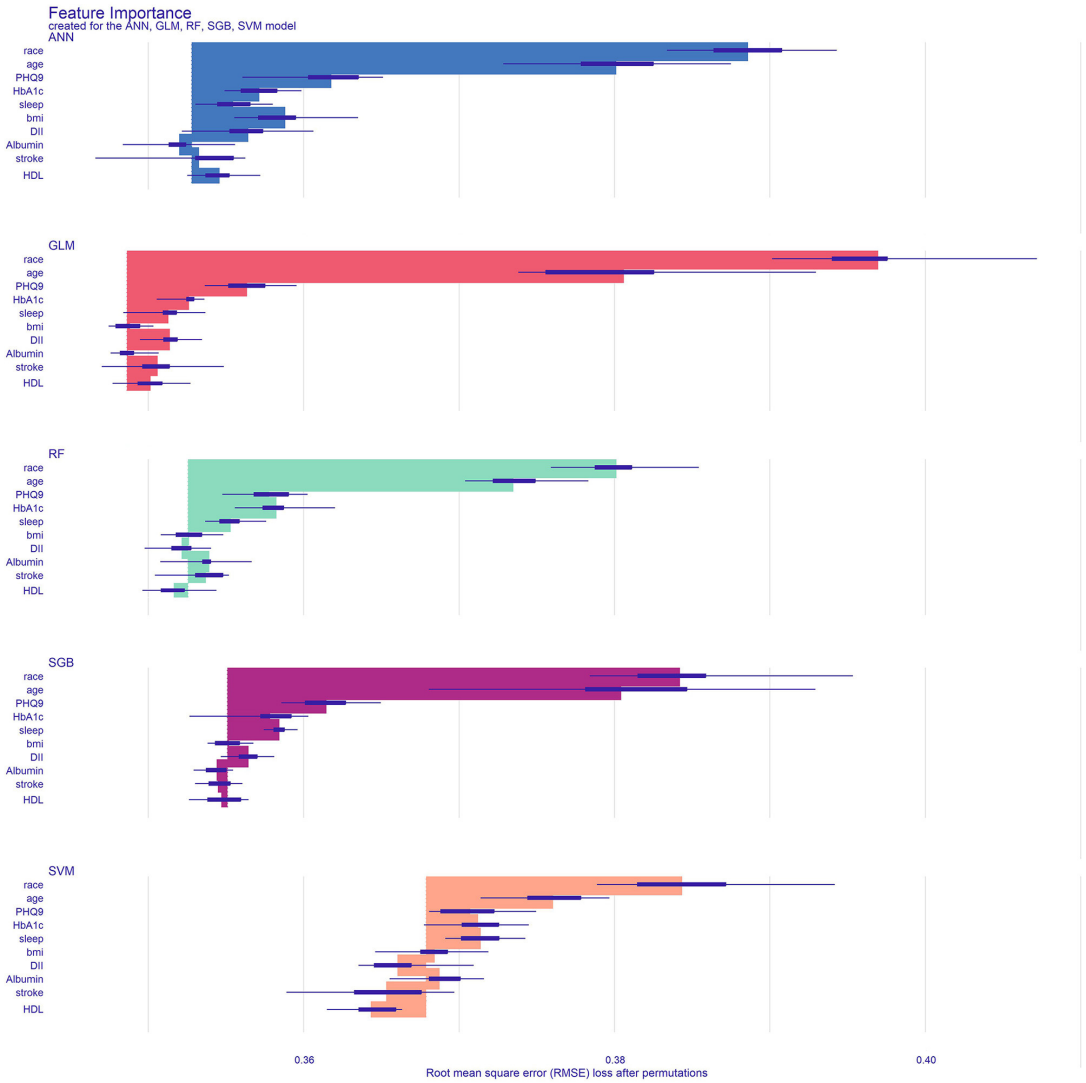
Analyze and visualize the feature importance of the classification model. GLM, generalized linear model; RF, Random Forest; SVM, support vector machine; ANN, artificial neural network; SGB, Stochastic Gradient Boosting; bmi, Body mass index; stroke, ever told you had a stroke; DII, Dietary Inflammatory Index; HbA1c, glycated hemoglobin; PHQ9, Patient Health Questionnaire-9; sleep, how much sleep do you get; HDL, direct HDL-cholesterol level.

The importance analysis of various factors shows that GLM is the best performance machine learning model in Figure 5. The 10 variables in order of importance are race, age, PHQ-9 score, HbA1c, sleep duration, BMI, DII, albumin, stroke history, and direct HDL-cholesterol level.

## Discussion

This study found that race, age, PHQ-9 score, HbA1c, sleep duration, BMI, DII, albumin, stroke history, and direct HDL-cholesterol level are considered important determinants of cognitive function in the elderly population of the United States. Race and age are the strongest predictors, followed by PHQ-9 score, HbA1c, and sleep duration, respectively. In addition, BMI, DII, albumin, stroke history, and direct HDL-cholesterol level are also predictors of cognitive impairment. Our results indicate that the GLM model predicts cognitive impairment in older adults better than the other four models.

There are many risk factors for the development of cognitive impairment in older adults; for example, excessive sleep duration increases the risk after adjusting for numerous relevant risk factors (Yuan et al., 2022a), and those with a low BMI (< 23 kg/m^2^) have a higher risk of developing dementia (Yuan et al., 2022b). With the advent of an aging society, cognitive impairment occurrence will become more common (Afzal et al., 2014). Many risk factors that affect cognitive function can be avoided and modulated, so it is of great interest to develop a simple and effective model for predicting the risk of cognitive impairment in older adults. The model screens for independent risk factors for cognitive impairment in older adults and also predicts the weight of each risk factor, which will facilitate the development of effective prevention strategies and recommendations in public health to prevent or delay cognitive impairment in older adults.

This study found that race and age are risk factors for the development of cognitive impairment in older adults, and a review of previous studies revealed a negative correlation between age and cognitive function (Lacreuse et al., 2020). Some studies that used MRI concluded that age-related decline in cognitive function was associated with changes in the integrity of the prefrontal area (Raz et al., 2005). Studies performed on animals found that age-related changes in the white matter and more-subtle changes in neurotransmitters, synaptic density, and neuron firing levels may underlie age-related declines in cognitive function (Sherwood et al., 2011). Race has also been found to be a risk factor for cognitive impairment in older adults; the APOE genotype has been found to be associated with cognitive impairment, and given the variations in APOE genotypes by race, especially between individuals of European and African ancestry, race plays an important role in this association and thus leads to the probability of cognitive impairment differing between races (Kim et al., 2017). In the present study, age and race had higher weights in the prediction model, and community and health-care units can rationalize medical resources based on this result when allocating resources.

Another risk factor for cognitive impairment in this study was the PHQ-9 score. A review of previous studies suggested that most findings for older adults have consistently supported higher levels of depressive symptoms being a key risk factor for cognitive deficits. This was consistent with our findings, with this adverse effect being particularly pronounced in older adults, who should therefore pay extra attention to depressive symptoms. At the neurocognitive level (Duman et al., 2016), depression is known as impaired cognitive flexibility and prefrontal inhibition disorder (Disner et al., 2011), which negatively affects cognition (Beck and Bredemeier, 2016). Impaired neuroplasticity is the theoretical basis of depression, which results in cognitive impairment, and patients with depression should therefore be actively treated medically and psychologically so as to reduce the risk of cognitive impairment.

In this study, HbA1c was also a risk factor for cognitive impairment among the older adults, and a review of previous studies found that patients with type 2 diabetes mellitus (T2DM) had poor cognitive performance (Biessels et al., 2014), and that higher HbA1c level is an independent risk factor for T2DM (Biessels et al., 2014). A higher HbA1c puts the body in a state of chronic inflammatory damage, and longer durations of diabetes and lower blood glucose levels cause progressive neuron damage (De Felice and Ferreira, 2014). Older adults should therefore strictly control their HbA1c levels in order to preserve cognitive function.

Prolonged sleep time may be one of the clinical predictors of a higher risk of cognitive impairment. Epidemiological studies have shown that there is a non-linear relationship between sleep time and cognitive function (Hou et al., 2020). In our study, we observed that sleep duration is an independent predictor of cognitive impairment. Previous studies have shown that patients with cognitive impairment sleep longer than older adults without cognitive impairment. Recent studies have also shown that prolonged sleep is an early marker of neurodegeneration (Westwood et al., 2017). The mechanism of prolonged sleep in dementia patients may be related to changes in the brain's sleep and wake-up regions, including the suprachiasmatic nucleus between the pineal gland and retina (Mihardja et al., 2020). Therefore, professional advice should be given on the control of sleep time for older adults. Both too short and too long sleep can lead to cognitive impairment. Doing a good job of education and maintaining good living habits will be beneficial to cognitive health.

BMI is another independent risk factor for cognitive impairment. Potential mechanisms for the pathophysiological relationship between BMI and AD risk include neuropathological changes occurring in regions such as the hypothalamus, which play a key role in regulating energy metabolism and food intake (Loskutova et al., 2010). As a modifiable factor, BMI may be a potential intervention for cognitive impairment.

DII is also a risk factor for cognitive impairment in the older adults. A review of previous studies indicated that some inflammatory molecules can cross the blood–brain barrier and increase neuroinflammation, thereby impairing cognitive function (Heneka et al., 2015), which is the neurological basis for cognitive impairment (Leng and Edison, 2021). It is recommended to strictly control the intake of foodstuffs associated with inflammation in the older adults.

The next risk factor identified in this study is albumin level, which is an essential nutrient for normal body function (Wu, 2016). A low protein intake may increase the risks of sarcopenia and frailty especially in the older adults, which are strongly associated with the development of cognitive impairment (Chang et al., 2016). Proline-rich peptides exert a preventive effect on dementia progression (Bilikiewicz and Gaus, 2004) and decreased serum protein affects the protective effect (Van De Rest et al., 2013), and so the older adults should consider protein supplementation in order to delay and reduce the risk of cognitive impairment.

This study found that a history of stroke is also a risk factor for cognitive impairment in older adults, and a review of previous studies suggested that cognitive impairment is common after stroke (Kwakkel et al., 2006) and can be caused directly by a stroke lesion or by structural and functional impairments resulting from the lesion (Carrera and Tononi, 2014). Stroke survivors also suffer from small-vessel disease and neurodegenerative disorders (Arba et al., 2017; Georgakis et al., 2019), which are the neurological basis for the development of cognitive impairment. Extra care such as secondary prevention should therefore be taken to prevent the development of cognitive impairment, even in those with no history of stroke.

Direct HDL cholesterol level is also a predictor of cognitive impairment. Studies of the former have shown that HDL has a positive impact on general cognitive performance in older adults. HDL cholesterol is used to remove excess cholesterol from cells and transport it back to the liver for bile processing, thus preventing atherosclerosis and protecting arteries (Félix-Redondo et al., 2013; Castañer et al., 2020). Low HDL is associated with decreased hippocampal volume, a particularly vulnerable region of the brain associated with neurodegenerative diseases (Hillbrand and Spitz, 1997). Therefore, HDL is known as cholesterol that is beneficial to the body (Hillbrand and Spitz, 1997). Many factors affect cholesterol metabolism, including lifestyle and behavioral factors, so it is possible to regulate cholesterol levels through lifestyle interventions. In summary, the mechanism of cognitive impairment is very complex. Currently, primary prevention is the most effective intervention method to prevent the occurrence of cognitive impairment. Through the above factors in this study, it can help doctors and potential patients achieve early intervention, prevention, and treatment combination, and reduce the occurrence of cognitive impairment.

### Limitations

This study was subject to some limitations. First, its cross-sectional design meant that data could not be collected strictly according to our specific requirements. Second, we did not identify the cause of any impairment. Third, this study was internally validated, and so external validation should also be conducted to determine whether the results can be generalized to wider populations and regions. Finally, we lack data on family history and hope to consider more comprehensive and more variables for further analysis in future research.

### Conclusions

ML model can become a reliable tool for predicting the occurrence of cognitive impairment in older adults. Among all prediction models, the GLM model is the most effective model, which can help clinicians accurately manage and early intervene in older adults at risk of cognitive impairment to reduce mortality.

## Data availability statement

Publicly available datasets were analyzed in this study. This data can be found here: https://www.cdc.gov/nchs/nhanes/index.htm~NHANES.

## Ethics statement

Ethical review and approval was not required for the study on human participants in accordance with the local legislation and institutional requirements. The patients/participants provided their written informed consent to participate in this study.

## Author contributions

Conceptualization: WL, SY, and WZ. Methodology and data curation: WL. Software: YS. Validation: WL and SY. Writing—original draft preparation: WL, LZ, and ZC. Writing, review, and editing: WL and WZ. Researching designs and revising manuscripts: LJ and ZC. All authors have read and agreed to the published version of the manuscript.
